# Spontaneous Distal Extrusion of Kuntscher Intramedullary Nail

**DOI:** 10.1155/2021/5587325

**Published:** 2021-10-18

**Authors:** W. B. Teh, R. Kuharajan, A. H. Noor Shafika, I. G. Nur Azhani

**Affiliations:** ^1^Department of Orthopaedics Surgery, Hospital Sultanah Nur Zahirah, Terengganu, Malaysia; ^2^Department of Orthopaedics Surgery, Hospital Kemaman, Terengganu, Malaysia; ^3^Department of Orthopaedics Surgery, Hospital Selayang, Selangor, Malaysia

## Abstract

Migration of femoral Kuntscher intramedullary nail (K-nail) proximally or distally within the femoral medullary cavity is a well-known documented complication, but spontaneous extrusion of a distally migrated K-nail is a rare complication. This is a case report of a 33-year-old lady who complained of sudden onset left knee pain and presented with spontaneous extrusion of a Kuntscher nail 12 years postinsertion. She underwent a successful K-nail removal surgery and showed a good clinical outcome after 6 months of outpatient follow-up.

## 1. Introduction

Kuntscher cloverleaf nail (K-nail) with its longitudinal slot is a type of intramedullary (IM) rod that was introduced by Gerhard Kuntscher working with Professor Fischer and Engineer Ernst Pohl at the University of Kiel in Germany in the 1930s [1, 2]. This stainless steel intramedullary nail was used for fixing simple transverse, short oblique, or Winquist-Hensen types I and II comminuted midshaft diaphyseal fractures of the femur [1]. The original nail was in the shape of the letter V, but later introduced in the four-leaved clover form for additional strength and easier use. Migration of femoral Kuntscher nail proximally or distally is a well-known documented complication, where distal migration of K-nail was reported as early in 1942s as one of the complications. But spontaneous extrusion of the K-nail through the knee joint is a rare complication.

The common etiologies of the extrusion are infection, delayed union with shortening, inappropriate K-nail size, disuse osteoporosis, and premature weight bearing. To avoid the possibility of late onset migration and spontaneous extrusion, it is recommended that K-nails be routinely extracted as soon as union and consolidation of the fracture are radiologically established [1]. We present a case of left knee arthropathy resulting from distal migration Kuntscher intramedullary nail secondary to chronic osteomyelitis.

## 2. Case Report

A 33-year-old lady, with underlying retroviral disease, presented with left knee pain for the past 2 years. She gave a history of a motor vehicle accident 12 years prior, where she sustained a closed left femur shaft fracture and was treated with a K-Nail. She was well; until 2 years ago, she noticed an increasing pain in her left knee, as well as a gradual reduction in range of motion. Pain was dull, aching, continuous, and progressively increasing in intensity causing limping and inability to squat easily. It was aggravated by sitting and standing up but relieved by nonsteroidal anti-inflammatory drugs.

**Figure 1:**
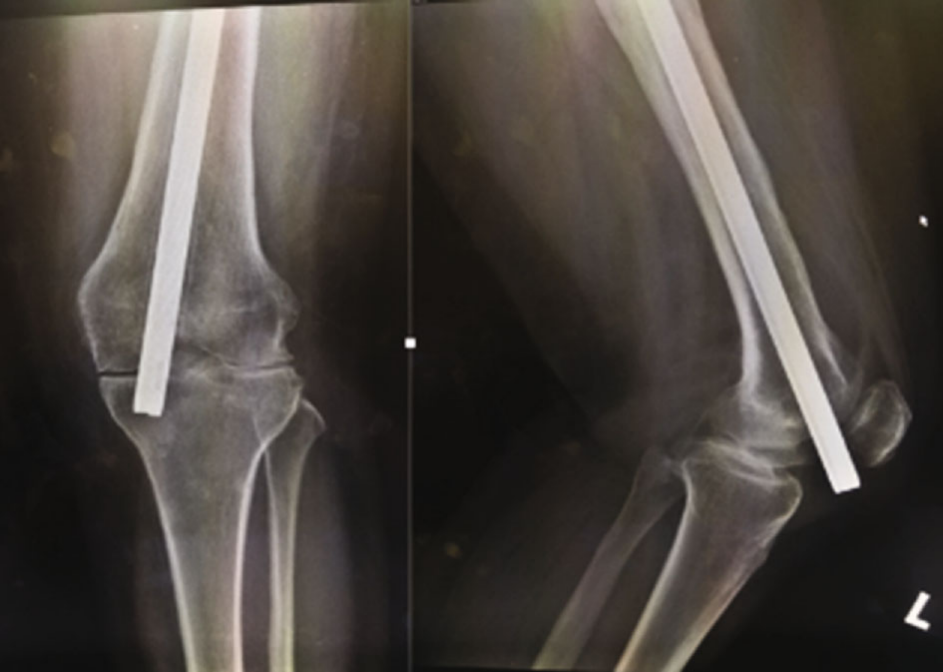
Plain radiograph shows the nail protruded through distal femur.

**Figure 2:**
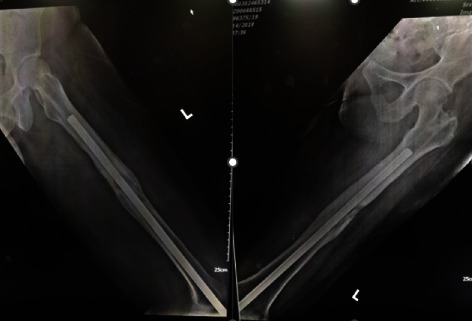
Plain radiograph shows the feature of chronic osteomyelitis over femur shaft.

Knee examination reviewed tenderness along the patellar as well as the knee joint line. There were no signs of active infection, and the patella was naturally mobile. Active knee range of motion (ROM) was 70°–80°, but passive ROM was 80°–90° but painful. There was no distal neurovascular deficit. Plain radiography confirmed the distal migration of Kuntscher nail into the left knee (Figure 1) as well as fracture union, but features suggestive of intramedullary chronic osteomyelitis (Figure 2) as well.

**Figure 3:**
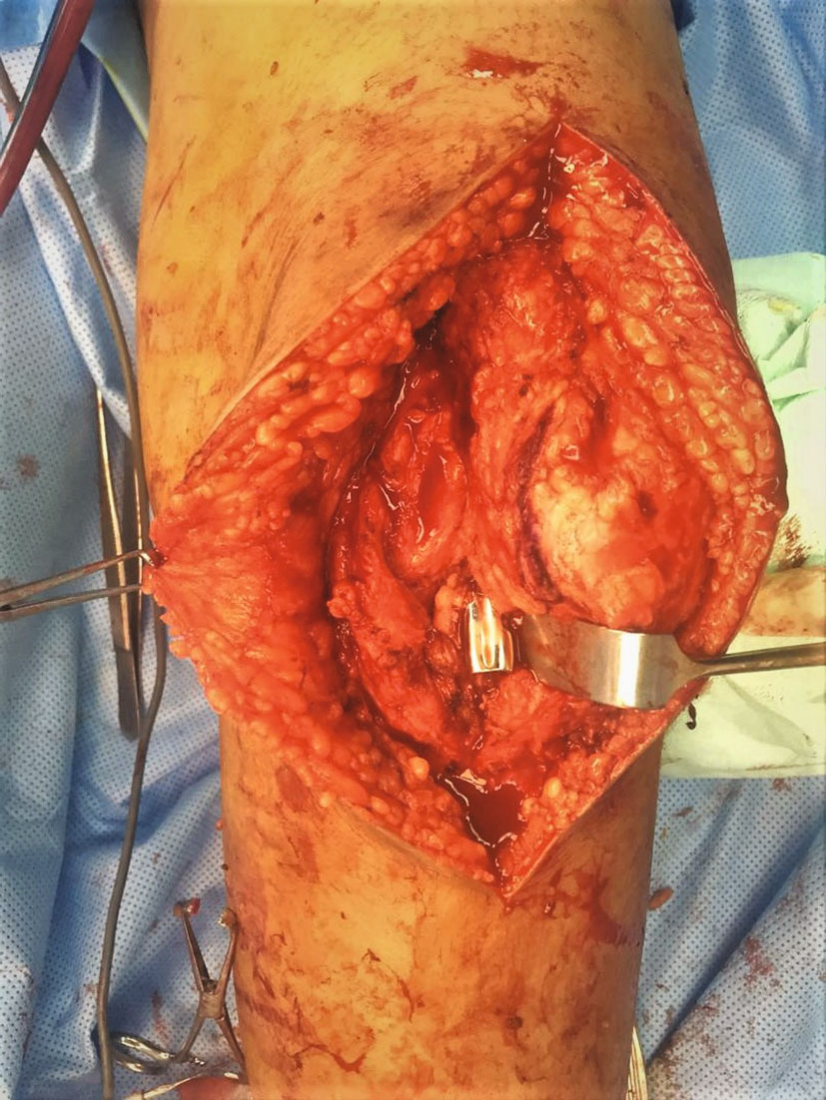
The protruded nail through medial femoral condyle, after debridement.

**Figure 4:**
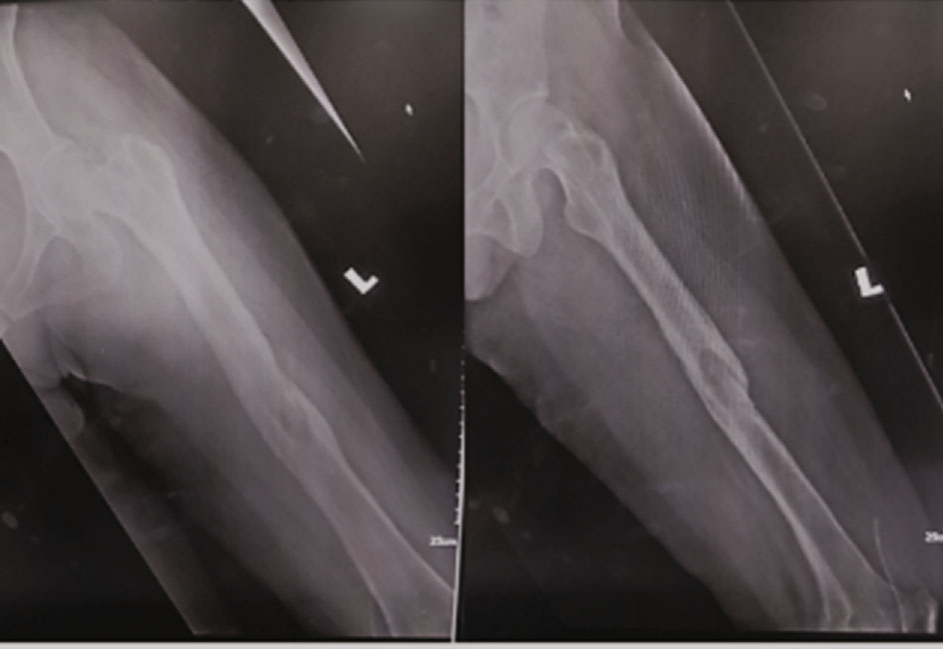
X-rays of left femur post K-nail removal.

**Figure 5:**
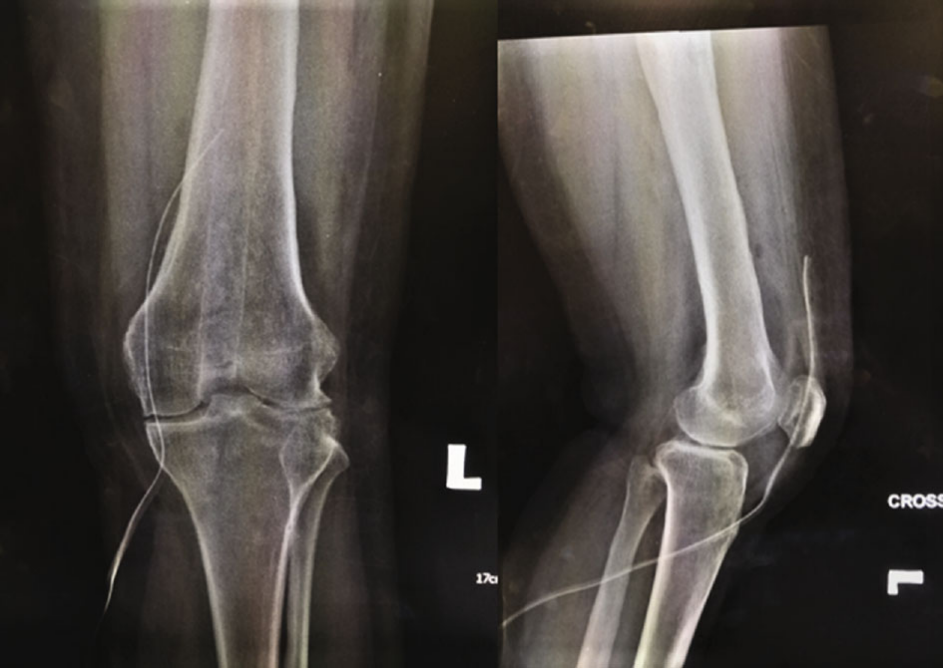
X-rays of left knee post K-nail removal.

**Figure 6:**
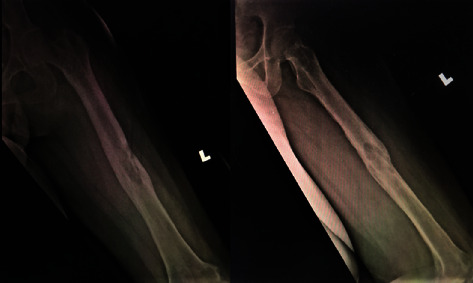
X-rays left femur at 6 months follow-up.

She underwent removal of the K-nail, utilizing a new anterior medial parapatellar approach incision via the knee (Figure 3). Intraoperatively, there was abundant fibrous tissue, which was debrided from the intra-articular surface of the knee and the cartilage of the medial femoral condyle was friable around the extrusion site. There were biofilm slime materials around the cloverleaf-shaped nail as well. Postoperative radiograph revealed the area of lucency at the healed fracture site, removed nail track, and osteopenic bone (Figures 4 and 5). Intraoperative cultures tested positive for Klebsiella pneumonia organisms where she was started on antibiotics for 6 weeks. Physiotherapy was scheduled for 2 weeks postoperatively; nonweight bearing was prescribed for 6 weeks. Postoperatively, the knee passive flexion improved to >110°, and during follow-up at 6 months, the patient was ambulating well without any complaint. Laboratory investigations at 6 months showed significant reduction in inflammatory markers as erythrocyte sedimentation rate (ESR) of 120 mm/hours to 2 mm/hours and C-reactive protein (CRP) of 24 mg/L to 3.2 mg/L. Plain radiography (Figure 6) at 6 months is still suggestive of intramedullary chronic osteomyelitis without any new pathologic fracture seen. In view of asymptomatic and patient knee flexion which was >110°, we decided to manage conservatively for the cartilage damage and continue monitoring for any complication arise in the future.

## 3. Discussion

In the era where the treatment of fractures of the femur is limited to traction or cast splintage, Kuntscher nailing becomes the Gold standard for femoral fracture. However, the pitfalls with K-nails are the failure to prevent migration, collapse, or rotation of the fractured fragments, especially in inherently unstable fractures [2]. Since the introduction of the concept of “locking” using bolts at each end of the modern nail, open Kuntscher nailing is no longer the common method of fixation of femoral shaft fractures.

The underlying etiologies and pathogenesis of the extrusion are subject to controversy and speculation where common etiologies are infection, delayed union with shortening, inappropriate K-nail size, disuse osteoporosis, and premature weight bearing. The reason for distal migration in our case may be due to infection with underlying comorbid along with delayed union with shortening, but the reason for the knee involvement may be iatrogenic. The presentation by our patient 12 years postinsertion was not different from that reported by other authors whose patients presented between 8 months and 5 years postinsertion [3, 4]. Most of the cases reported by De Belder [4] had proximal half fractures involving the isthmus; only one had lower most femoral fracture. Most literature reviewed distal migration is more common with proximal femoral shaft fractures, the same as the index case. The literature reviewed does not indicate which fracture configuration is prone to migration [4–6]. The presence of the K-nail for a long time (6 years) may have also given rise to products of ionization and foreign body reaction which accumulated between the nail and the bone to cause a positive expulsion of the nail [2]. Early extraction as soon as union and consolidation of the fracture are radiologically established is advised to prevent the possibility of late onset migration and spontaneous extrusion of K-nail.

## 4. Conclusion

Distally migrated K-nail should be highlighted and taken into consideration by young and upcoming surgeons as one of the causes of knee arthropathy.

## Data Availability

We have acquired consent from the patient for all photographs of the patient's body parts and imaging to be used in publication purpose.
